# Supplementary material for: Are carbon emissions trading and green financial instruments synergistic? -Comprehensive quantitative research based on content analysis
Source: PLoS One. 2024 Mar 7;19(3):e0298601. doi: 10.1371/journal.pone.0298601 (PMC10919601; doi:10.1371/journal.pone.0298601)
Supplement: S1 Data — (ZIP) [file pone.0298601.s001.zip › Data/地级市Z_F工作报告.zip!/░▓╗╒/╕╖╤⌠╩╨/└┤╫╘╣1⁄2╓┌║┼í╛┬φ┐╦╩2╛▌═°í┐_2016.docx]

各位代表：

现在，我代表市人民政府，向大会报告政府工作，请予审议，并请市政协委员和其他列席人员提出意见。

一、过去五年工作回顾

过去的五年，在省委、省政府和中共阜阳市委的正确领导下，全市上下认真贯彻党的十八大和十八届三中、四中、五中全会精神，深入学习习近平总书记系列重要讲话精神，科学把握大势，积极主动作为，奋力攻坚克难，较好完成了“十二五”确定的主要目标任务，在全面建成小康社会征程中迈出了坚实的步伐。

（一）国民经济较快发展，综合实力大幅提升。全市生产总值突破千亿元大关，达到1267.4亿元，“十二五”期间年均增长10.3%；财政收入突破200亿元，年均增长17.8%；经济结构不断优化，三次产业结构由27.3：39.2：33.5调整为22.6：41.5：35.9；规模工业增加值超过500亿元，年均增长15.4%；农业生产实现“十连增、十二连丰”，粮食总产连续八年超百亿斤，荣获“全国粮食生产先进市”称号；社会消费品零售总额达到674.7亿元，年均增长15.2%；城镇、农村常住居民人均可支配收入较快增长，分别达到23450元和9100元。

（二）项目建设成效显著，发展后劲明显增强。累计完成固定资产投资3375亿元，是“十一五”的3.1倍。工业投资持续加大，华鑫铅业、天能电池、昊源化工航天炉和欣奕华新材料等重点项目建成投产。省级开发园区发展到11家，面积扩大到233平方公里，工业增加值占全市的70%以上，成为工业经济发展的主战场。商贸市场加快建设，临沂商城、阜阳汽贸物流园、颍州万达广场建成开业。交通、水利基础设施建设明显加快，建成国省干线公路297公里，市县快速通道全面建成通车，阜新、阜六高速和阜六铁路建成运营，漯阜铁路电气化改造完成，商阜杭高铁开工建设，阜阳机场开通航线11条、年旅客吞吐量突破40万人次。完成了行蓄洪区安全建设、大中型涵闸除险加固和重点中小河流治理工程，阜阳船闸、颍上船闸、耿楼船闸建成，沙颍河航道全线复航，颍州港投入使用。

（三）城乡建设步伐加快，人居环境显著变化。新型城镇化全面提速，阜城中心城市、县域中小城市、特色乡镇、美丽乡村“四位一体”发展格局初步形成，全市常住人口城镇化率达到38.8%，比“十一五”末提高6.9个百分点。阜城建设力度加大，五年投入资金500亿元，是“十一五”的12.5倍，建成区面积由76平方公里扩大到120平方公里，常住人口由70万人增加到120万人。城南新区累计投资112亿元，完成一期主干路网建设，综合管网、环卫设施、园林绿化同步实施。棚户区三年改造计划扎实推进，累计改造1173万平方米。建设改造了阜颍路、三清路、淮河路、颍州路和北三环路等主干道路，颍上路泉河大桥建成通车，东环路、向阳路颍河大桥开工建设。城市公共交通快速发展，连续四年每年新添置100辆空调公交车。引进建成公共自行车系统，受到市民的普遍欢迎。以“三项整治”为抓手，城市管理逐步步入规范化轨道，城市内涵有效提升，先后被列为省级文明城市、安徽园林城市和国家智慧城市、信息惠民试点城市。阜城变大了、变高了、变美了，城市首位度、美誉度和辐射力大为提升。五县城建设步伐加快，承载能力进一步增强。中心镇建设取得实效，美丽乡村建设扎实推进。森林增长工程深入实施，成片造林50万亩，建成农田林网315万亩。城乡面貌的有效变化，提升了阜阳形象，促进了整体发展，也提振了干部群众的“精气神”。

（四）改革开放实现突破，发展活力有效激发。行政体制改革力度加大，累计精简行政审批事项63.3%，成为全省行政审批较少的市。政府权力清单、责任清单和涉企收费清单全面推行，工商、质监、食品药品监管体制改革顺利完成。财税金融改革步伐加快，政策性投融资担保体系不断完善，8家农商行成功组建，五县市设立了村镇银行，交通银行、光大银行入驻阜阳。医药卫生体制改革扎实推进，县级公立医院改革全面开展。科技创新取得突破，新增高新技术企业70家，发明专利授权量达到571件。招商引资深入推进，引进市外资金3734亿元，年均增长26%。对外开放步伐加快，实现进出口总额62.2亿美元，年均增长33.3%。对外交流不断扩大，先后与美国加州蒙特利公园市、澳大利亚肖尔黑文市结为友好城市。

（五）社会事业全面进步，民计民生持续改善。民生工程不断拓展提升，五年投入资金432.7亿元，是“十一五”的4.6倍，惠及800多万城乡居民。扶贫开发取得实效，累计减少贫困人口99.2万人，完成378万农村人口安全饮水工程。高度重视就业再就业工作，持续开展“接您回家”系列活动，新增城镇就业37万人，转移农业富余劳动力70万人。城乡基本养老、基本医疗保险制度和大病保险实现全覆盖。教育设施和医疗卫生两个“三年行动计划”基本完成，累计投入46.5亿元。全市新建学校126所、医院24所，看病难、看病贵和学校大班额现象得到有效缓解。精神文明创建活动扎实开展，当选全国道德模范、“中国好人”、“安徽好人”位居全省前列，高思杰被中宣部授予“时代楷模”。文化惠民工程深入实施，认定国家级非物质文化遗产9个，荣获“中国书法城”称号。成功举办了第14届亚洲跳伞锦标赛暨国际跳伞公开赛和市第十二届运动会，阜阳籍运动员邓琳琳荣获伦敦奥运会体操金牌。八里河景区被评定为国家AAAAA级旅游景区，颍上迪沟、太和沙颍河等5处湿地被列入国家湿地公园。“颍淮蓝天工程”稳步推进，秸秆禁烧和综合利用工作成效明显，空气质量位居全省前列，入选国家循环经济示范城市。安全生产形势逐步向好，连续七年未发生重特大安全事故。

（六）依法行政扎实推进，自身建设不断加强。大力推进法治政府建设，切实规范政府系统重大事项决策行为，努力拓展政务公开，“六五”普法任务圆满完成。持续开展政风行风评议和市直单位百名科长考评活动，政府职能加快转变，行政效能不断提升。自觉接受人大法律监督，依法执行人大决议决定，办理人大代表议案建议565件。主动接受政协民主监督，办理政协委员提案1281件。认真接受司法监督，健全行政机关负责人依法出庭应诉制度。认真开展群众路线教育实践活动和“三严三实”专题教育，大力开展“四风”整治行动。全面加强作风建设和廉政建设，严格实行督查考核，强化行政监察和审计监督，政府执行力和公信力进一步提升。

各位代表！刚刚过去的2015年是“十二五”收官之年，我们主动适应经济发展新常态，扎实做好稳增长、促改革、调结构、惠民生、防风险各项工作，有效保持了经济社会平稳较快发展。一年来，主要做了以下几个方面的工作：

一是全力以赴促进经济增长。统筹做大工业、做强农业、做活三产，着力实施调转促“576”行动计划，努力扩大经济总量、提升发展质量，主要指标增幅跃居全省前列。全年生产总值增长9.5%，高于全省平均水平0.8个百分点，比上年提升6个位次。工业经济较快发展，规模工业增加值增长10.8%，位居全省第4位，新增规模工业企业200家。实现战略性新兴产业产值228.6亿元，增长33.6%，太和现代医药产业基地入选国家级火炬特色产业基地和全省首批战略性新兴产业集聚发展基地。开发园区转型升级步伐加快，阜合现代产业园区发展势头持续向好，临庐现代产业园区开工建设。现代农业提质增效，土地流转面积达到483万亩，流转率56.2%，规模养殖比重达76%。新注册家庭农场2090家、认定市级以上龙头企业95家。实现农产品加工业产值764亿元，增长19%。商贸市场日趋繁荣，颍泉万达广场开工建设，宝龙城市广场、合肥百大购物中心和阜阳农产品交易中心加快推进。电子商务、物流快递等新兴业态快速发展，皖北快递产业园落户阜阳，青年电商园、阿里巴巴“特色产业带”和淘宝阜阳馆先后建成，电商主体发展到8000多家，网络交易额超过30亿元。银行保险业稳健运行，金融机构贷款余额1187亿元，增长23.5%。

二是千方百计扩大有效投入。切实把扩大投资作为加快发展的主要抓手，坚持经济工作项目化、项目工作责任化，倾斜领导、保证时间，加强调度、严格考核，投资规模不断扩大，全年完成固定资产投资1005亿元，增长24.8%，位居全省首位。一批重点投资项目取得突破，单体投资超30亿元的金种子生态产业园、江汽中重型卡车制造项目扎实推进，华润电厂二期项目得到核准，阜阳卷烟厂易地技改、意大利生物质产业园、阜阳煤基新材料项目进展顺利。交通、水利基础设施建设力度加大，分别完成投资54亿元和24亿元，比上年增长25.4%和71%。充分发挥招商引资主渠道作用，大力开展各类招商活动，全年引进市外资金1214亿元，增长25.2%，超额完成年度目标任务。

三是加大力度推进城乡建设。强力推进阜城建设与管理，全年完成投资206亿元，增长29%，创历史最高，位居全省第2位。全年新建城区道路110公里、改造150公里，西南大外环、东三环和城南新区路网、职教园区路网加快建设。园林绿化提升行动成效明显，完成重点绿化提升工程105处，新建城市游园30个，新增绿地面积85万平方米。切实加大征迁力度，全年完成征迁500多万平方米。有效实施主次干道楼体立面整治和亮化工程，强力推进拆违拆旧整治环境专项行动。大力开展县城“三治三增三提升”行动，五县市完成投资160多亿元。加强中心镇和美丽乡村建设，40个重点示范村基本建成，45个重点示范村加快建设，实现“三线三边”综合整治向全市延伸。

四是着力推动重点领域改革。全面取消非行政许可审批事项，深入推进“放管服”改革，市级行政权力精简37.9%，减少涉企收费近15亿元。机关公务用车制度改革全面实施，行业协会撤并工作顺利完成。商事制度改革加快推进，“三证合一”、“一照一码”全面实行，新增市场主体5.2万户，增长40%。临泉县、颍泉区农村综合改革试点工作稳步推进，土地确权颁证顺利开展。在全省率先实施户籍制度改革，实行两个月以来，新迁入中心城区人口3379人。财税金融改革稳步推进，全面开展预算评审，财政资金使用效益进一步提高。农村商业银行对县域经济发展的支撑作用明显增强，全年实现税收7.4亿元。大力发展普惠金融，在全省率先完成行政村农村金融综合服务站设立工作。“新三板”挂牌企业达到5家，省股权交易中心挂牌企业达到119家。积极推广政府与社会资本合作等融资模式，设立新型城镇化各类建设基金，募集资金近200亿元。华源纺织改制工作顺利完成，教育、卫生、文化等领域改革也都取得新的进展。

五是尽心尽力改善群众生活。大力推进基本公共服务均等化，33项民生工程任务全面完成，民生类支出达369亿元，占财政支出的85.6%。积极促进就业创业，全年新增1.6万人就地就业，近千人返乡创业。加快推进保障性安居工程建设，开工建设保障性住房80292套，基本建成31498套，占全省的20.2%，为我市历史之最，完成农村危房改造18400户。企业退休人员基本养老金人均月增197.3元，城乡居民基础养老金月标准提高到70元。完善社会救助体系，扶残助残、公益慈善事业取得新进步。阜阳烈士纪念馆建成开馆。公共文化体系建设得到加强，新建了一批乡镇综合文化服务中心和农民文化乐园。

六是切实维护社会和谐稳定。加快社会治理法治化进程，推行网格化新型社区管理模式，市级社会服务管理信息化平台建成使用。全面加强应急管理，灾害防御能力进一步提升。坚持一手抓诉求解决、一手抓依法治访，信访工作进步明显。强化食品药品安全监管，市县乡三级监管体系进一步完善。严格落实安全生产责任制，扎实开展“铸安”行动。严厉打击各类违法犯罪活动，全市禁毒整体战和临泉禁毒攻坚战取得良好效果，人民群众安全感持续上升，社会大局保持和谐稳定。认真做好武警、人防和民兵预备役工作，强武阜阳建设扎实开展。妇女、儿童及老龄、老干部工作进一步加强。民族、宗教、统计、文物、档案、地方志、地名、外事、侨务、港澳台等工作也都取得了新的成绩。

各位代表！过去的五年，是阜阳发展史上综合实力实现快速提升、城乡面貌发生重大变化、人民群众得到更多实惠的五年，成绩来之不易，必须倍加珍惜。这是省委、省政府和市委正确领导的结果，是市人大、市政协大力支持的结果，是全市上下团结一心、奋力拼搏的结果。在此，我代表市人民政府，向广大工人、农民、知识分子、干部、离退休老同志，向驻阜解放军指战员、武警官兵和政法干警，向各民主党派、工商联、无党派人士、各人民团体和社会各界人士，向所有关心支持阜阳建设与发展的同志们、朋友们，表示衷心的感谢！

在肯定成绩的同时，我们也清醒地认识到，我市经济社会发展中仍存在一些困难和问题。一是发展不足的问题依然突出，经济总量不大，人均水平偏低，工业化、城镇化水平不高，与全国、全省平均水平仍有较大差距；二是结构调整任务艰巨，战略性新兴产业规模不大，传统型产业比重偏高，现代服务业发展相对滞后，农业基础较为薄弱；三是民生保障能力有待提高，就业、教育、卫生、文化、社会保障等公共服务均等化水平较低，脱贫攻坚任务艰巨，环境保护、安全生产、食品药品安全等方面还存在不少问题。同时，一些地方和部门开拓创新意识有待增强，工作能力有待提升，少数干部为政不为、缺乏担当精神，“不严不实”现象还不同程度地存在，发展环境仍需进一步改善。对此，我们一定高度重视，采取有力措施，认真加以解决。

二、未来五年主要任务

“十三五”时期，是全面建成小康社会的决胜阶段，是我市必须大有作为而且能够有大作为的黄金发展期。根据市委《关于制定国民经济和社会发展第十三个五年规划的建议》，“十三五”发展的指导思想是：高举中国特色社会主义伟大旗帜，全面贯彻党的十八大和十八届三中、四中、五中全会精神，以马克思列宁主义、毛泽东思想、邓小平理论、“三个代表”重要思想、科学发展观为指导，深入贯彻习近平总书记系列重要讲话精神，认真落实中央和省委、省政府重大决策部署，按照“五位一体”总体布局和“四个全面”战略布局，坚持创新发展、协调发展、绿色发展、开放发展、共享发展，以提高质量效益为中心，以调转促“576”行动计划为抓手，全面加快发展步伐，坚决打赢脱贫攻坚战，努力把阜阳建设成为皖北区域性中心城市、“四化”协调发展先行区和安徽发展重要增长极，确保如期实现全面建成小康社会的目标。

“十三五”经济社会发展主要目标是：

——经济总量快速扩张。主要经济指标总量领先皖北，增长速度高于全省。到“十三五”末，全市生产总值超过2200亿元，财政收入超过350亿元。

——质量效益全面提升。自主创新能力显著增强，科技进步对经济增长贡献率明显提高，力争进入安徽省创新型城市行列。新型城镇化不断加快，常住人口城镇化率达到45%，户籍人口城镇化率达到35%。

——产业结构持续优化。调结构转方式促升级步伐加快，战略性新兴产业产值超过1000亿元，现代服务业增加值占服务业比重达到55%，三次产业结构趋向合理，基本建成特色鲜明的现代产业体系。

——人民生活日益改善。城镇、农村常住居民人均可支配收入增幅高于全省平均水平，全面完成脱贫攻坚任务，基本公共服务均等化追赶全省平均水平。群众生活质量和健康水平进一步提升，权益得到充分尊重和切实保障。

——生态环境明显变化。生产生活方式加快向低碳、绿色转变，环境质量进一步改善，争创国家园林城市、森林城市和文明城市。水、大气、土壤等污染得到有效整治，如期实现省下达的节能减排目标。

——文明程度显著提高。公共文化服务体系基本建成，文化事业和文化产业快速发展。城乡居民科学文化素养、法治意识和文明素质普遍增强，社会公德、职业道德、家庭美德、个人品德水平进一步提升。

——制度体系更加健全。重点领域和关键环节改革取得决定性成果，开放型经济新体制基本形成。人民民主不断扩大，法治政府建设步伐加快，依法治市全面推进，司法公信力明显提高。

“十三五”期间，突出抓好五大任务：

（一）坚定不移推进创新发展，大力促进经济转型升级。切实把创新作为加快发展的战略基点，全面加快转型升级步伐，努力培育发展新动力，提供发展新支撑。一是打造现代产业体系。着力实施战略性新兴产业发展工程，重点发展生物医药、装备制造、电子信息、节能环保、文化创意等产业。深入实施传统产业提升工程，推动绿色食品、机械制造、煤电化工、再生资源利用等产业向中高端发展。大力实施农业产业化推进工程，积极构建现代农业产业体系、生产体系和经营体系。统筹实施服务业加快发展工程，促进传统性服务业现代化、生产性服务业专业化、生活性服务业品质化。加快推进产业深度融合，努力形成以现代制造业为主导、现代农业为基础、现代服务业为支撑的现代产业体系。二是实施创新驱动战略。不断推动以科技创新为核心的全面创新，建立健全以企业为主体、产学研相结合的技术创新体系，大力引进大专院校、科研院所在我市设立分支机构，加快太和县省农科院皖北分院建设。支持企业创建科研平台，加快科技成果转化推广。不断深化科技体制改革，加大研发经费投入，构建有利于大众创业、万众创新的良好环境。三是深化重点领域改革。以正确处理政府与市场关系为核心，以解决发展中的突出问题和结构性矛盾为导向，深入推进行政体制、企业机制、财税金融和农业农村等领域事关全局的重点改革，全面激发市场和社会活力。

（二）坚定不移推进协调发展，加快形成整体联动格局。坚持在协调推进中拓宽发展空间，在厚植优势中激发发展动力，在补齐短板中增强发展后劲。一是统筹城乡协调发展。抢抓新型城镇化建设重大机遇，按照“四位一体”布局，突出阜城带动，着力扩大城市规模，完善主体功能，提升特色品位，建设大美阜城，着力打造具有重要影响力的区域性中心城市。统筹推进五县市组群式发展，围绕产城一体，加强基础设施建设，强化功能配套，推动县城健康有序发展。按照做大总量、突出特色、转型发展、提高效益的要求，立足区位条件和资源禀赋，发挥比较优势，着力壮大实力，走出一条各具特色的县域经济发展新路子。加强中心镇建设，围绕主要干道，打造一批特色集镇带。加快美丽乡村建设，建成一批生态宜居村庄美、兴业富民生活美、文明和谐乡风美的中心村。二是提升综合承载能力。以枢纽化、网络化、一体化为方向，加快建设以快速铁路、高速公路、高等级航道、民航机场为骨架，以普通铁路、公路、航道、城市轨道交通和农村公路为基础的现代综合交通体系，巩固提升阜阳交通枢纽城市地位。进一步完善管网、通讯、公厕、污水垃圾处理等基础设施，提升综合保障能力。三是加强精神文明建设。坚持用中国梦和社会主义核心价值观凝聚共识、汇聚力量，提升公民文明素养，打造“好人阜阳”品牌。深入开展群众性精神文明创建活动，不断提升文明行业、文明单位、文明村镇和文明社区创建水平。

（三）坚定不移推进绿色发展，全面加快生态建设步伐。大力实施生态立市、绿色发展战略，促进人与自然和谐共生，建设绿色美好家园。一是优化区域发展格局。落实主体功能区规划，逐步建立科学合理的城镇化格局、农业发展格局、生态安全格局。推动重点开发区域提高产业和人口集聚度，对重点生态功能区实行产业准入政策。以泉河生态经济示范带项目为引领，统筹推进淮河流域综合治理与振兴发展，打造区域发展新的增长极。二是加强生态保护和建设。实行最严格的环境保护制度，深入实施大气、水、土壤污染防治行动计划。坚持城乡环境治理并重，实现城镇生活污水垃圾处理设施全覆盖。严守资源环境生态红线，建立分工明确、统一协调的自然资源监测和统计体系。大力实施森林增长工程，创建一批森林城市、森林城镇、森林村庄。重视生态文明理念的传播，推动全社会形成绿色消费自觉。三是推动低碳循环发展。强化约束性指标管理，落实能源和水资源消耗、建设用地等总量和强度双控行动。深入推进国家循环经济示范城市建设，加快企业循环式生产、产业循环式组合、园区循环式改造，建设清洁低碳、安全高效的现代能源体系。

（四）坚定不移推进开放发展，不断拓展经济增长空间。深入实施开放引领战略，积极对接国家新一轮开放总体布局，全面提升开放型经济水平。一是积极融入国家和省重大战略。抢抓国家推进“一带一路”、中原经济区、淮河生态经济带建设以及省委、省政府振兴皖北的重大机遇，提升基础设施和市场体系互通对接能力，积极参与区域分工合作，以大开放推动大发展。二是切实加快开发园区建设步伐。坚持创新集聚、绿色集约、产城融合、示范带动，促进开发园区由粗放扩张型向质量效益型转变。突出产业特色，培育一批主业突出、特色鲜明、核心竞争力强的开发园区。深入推进合作共建，提升专业化开发水平，力争两个以上开发园区升级为国家级园区。三是全面提升招商引资工作水平。坚持把对外开放作为第一战略、招商引资作为第一抓手，不断创新招商引资方式，引导市外资金重点投向战略性新兴产业、先进制造业、现代服务业、现代农业等领域，主动承接境内外加工贸易转移，争创国家级加工贸易梯度转移示范基地。大力发展外向型经济，加强通关便利化建设，积极创建国家一类陆路口岸、皖西北保税物流中心，加快形成内外联动的全面开放新格局。

（五）坚定不移推进共享发展，努力提升人民群众福祉。按照人人参与、人人尽力、人人享有的要求，更加注重机会公平，以民生工程为抓手，着力解决事关人民群众切身利益的突出问题，不断提升基本公共服务均等化水平。一是大力推进扶贫开发。按照“三年集中攻坚、两年巩固提升”的要求，深入实施脱贫攻坚工程，以增加贫困群众收入、改善贫困地区生产生活条件为重点，大力实施水、电、路“三项工程”，围绕“六个精准”、“五个一批”，确保2018年实现贫困村自来水“村村通”，完成道路畅通工程建设任务，基本解决贫困地区农村电网“卡脖子”等突出问题。到2020年，64.3万贫困人口全部脱贫，扶贫开发重点县全部“摘帽”。二是优先发展教育事业。加快发展学前教育，全面普及15年基础教育，办好特殊教育，促进义务教育均衡发展。构建现代职业教育体系，推进职教大市、技工大市建设。加强教师队伍特别是乡村教师队伍建设，改善贫困地区薄弱学校基本办学条件，实现家庭经济困难学生资助全覆盖，促进教育公平。三是切实完善就业服务和社会保障体系。实行更加积极的就业政策，深入推动大众创业工作，大力发展劳动密集型产业和吸纳就业能力强的中小企业，创造更多就业岗位。健全就业服务体系，落实就业援助制度，保障职工和企业合法权益。持续增加城乡居民收入，多渠道增加居民财产性收入。建立更高水平的社会保障体系，认真做好医疗、工伤、失业保险省级统筹工作，确保困难群众基本生活。四是着力推进健康阜阳建设。建立覆盖城乡的基本医疗卫生制度和现代医院管理制度，构建布局合理、分工协作的医疗服务体系和分级就医格局。实施食品安全战略，形成严密高效、社会共治的食品安全治理体系。开展应对人口老龄化行动，推进医疗卫生与养老服务结合，鼓励各类市场主体增加养老服务和产品供给，更好保障老有所医、老有所养。

三、今年重点工作安排

今年是全面实施“十三五”规划的开局之年，也是推进结构性改革、实施调转促“576”行动计划的关键一年。经济社会发展主要预期目标是：全市生产总值增长9%，财政收入增长10%，固定资产投资增长20%，规模工业增加值增长11%，社会消费品零售总额增长11%，城镇、农村常住居民人均可支配收入分别增长9%和10%，居民消费价格涨幅控制在3%左右，城镇登记失业率控制在4%以内，完成省下达的节能减排任务。

为实现上述目标，重点做好八项工作：

（一）突出三产联动，大力促进经济较快发展。坚持把发展实体经济作为经济工作的主体和核心，着力推进三产融合、协同发展。一是加快实施工业突破。坚定不移实施工业强市战略，坚持大小结合、内外结合、土洋结合，多措并举促进工业经济平稳较快发展。围绕做大工业总量，一手抓好增量扩大，加强招商引资和对接合作，大力引进一批品牌企业，力争完成工业投资420亿元；一手抓好存量扩张，挖掘企业潜力，实施技术改造项目172个，其中亿元以上项目68个，促进企业提质增效。围绕做优产业体系，继续推进工业企业质量提升行动计划，重点推进太和现代医药、界首新能源和临泉电子信息等战略性新兴产业基地建设，改造升级农副产品加工、煤电化工和纺织服装产业，着力壮大食品、烟草、酿酒等传统产业。稳妥处置“僵尸企业”，实施企业减负行动，支持企业加快发展。围绕做强园区平台，加强基础设施建设，创新管理制度，提升集约化经营水平，加快开发园区转型升级步伐。二是加快发展现代农业。大力开展粮食绿色增产模式攻关，依靠科技、主攻单产、提升品质。深入推进农业结构调整，扩大高效经济作物面积，引导阜城三区率先加快结构调整，大力发展都市农业、城郊农业、休闲农业和观光农业。加快推进农村一二三产业融合发展，扶持壮大龙头企业，促进农副产品就地加工转化，推动“百亿粮仓”向“千亿厨房”转变。加快培育新型农业经营主体，支持专业合作社拓宽服务领域。加强高标准农田建设，推广农业物联网、农业光伏等新技术应用，促进农业机械化全程全面发展，增强农业综合生产经营能力。三是加快提升现代服务业水平。围绕建设国家区域流通节点城市，加快阜阳农产品物流中心、国际服装城和临泉义乌小商品市场等重点项目建设，新上一批大市场、大商场、大物流项目。大力发展工业设计、科技服务等生产性服务业，积极发展健康养老、体育健身、职业培训等生活性服务业。加快颍泉公铁水物流园、太和发艺产业园、阜南柳木文化产业园建设。实施外贸企业孵化工程，加强出口示范基地建设，不断提高经济外向度。

（二）突出项目带动，持续扩大有效投入规模。切实把项目投资作为经济发展的“牛鼻子”，充分发挥投资对经济增长和结构优化的关键作用。一是认真抓好项目编制。围绕政策导向，立足资源禀赋，瞄准投资需求，重点在产业升级、基础设施、生态环保、民生改善等领域，谋划一批打基础、管当前、利长远的重大项目，全面提升项目编制科学化水平，为项目争取奠定良好基础。二是大力推进项目转化。注重加强与国家产业政策的有效对接，积极开展项目申报，加大争取力度，着力提高成功率，力争更多项目进入国家和省计划的盘子。注重把项目推向招商引资主战场，大力开展专业招商、驻点招商、精准招商和以商引商，确保全年引进市外资金增长20%以上。注重把项目推上社会资本的舞台，认真研究财税金融政策，加强政银企合作，促使金融机构扩大信贷规模。着眼激活民间资本，推出一批更有吸引力的项目，加强与社会资本合作对接，大力调动民间投资的积极性。三是强力推动项目落地。对今年重点实施的689个项目，实行市县领导联系制度，强化“四督四保”推进机制，着力解决征地拆迁、资金调度、环境保障等问题，争取早开工、早竣工、早见效。加快重点项目建设步伐，全力支持商阜杭、郑合高铁建设，大力推进高铁站新区建设。开工建设阜阳南北大通道，实施阜阳至利辛、颍上至凤台、阜南至淮滨国省道改建改善工程。抓紧做好阜阳—霍邱—合肥、阜阳—淮滨和沿淮高速等3条高速公路前期工作。建成颍上复线船闸、临泉杨桥船闸，实施汾泉河航道整治工程，积极做好淮河南照阜阳港项目前期工作，大力推进引江济淮工程建设。

（三）突出统筹协调，全面加速城乡建设进程。按照城乡一体化发展的要求，加快构建以阜城为核心、以五县城为联体的城市群。一是做大城市规模。今年阜城安排建设项目551个，总投资420亿元，确保完成年度投资260亿元以上。重点推进城南新区建设，在全面加快18平方公里一期工程建设、完善配套设施的同时，科学编制规划，加快推进二期建设。适度扩张颍州、颍泉、颍东建设规模，拉大城市框架。支持五县市加强规划建设管理，深入推进“六项行动”，不断强化产业支撑，有效增强城市承载能力。全面落实户籍制度改革实施方案，加快农业转移人口及常住居民市民化。二是完善城市功能。进一步加强阜城道路、电力、供水、供气和地下综合管廊建设，加快“三馆一院”建设进度，推进社区文化体育活动中心建设。大力推进公交场站及客运枢纽建设，年内新添置100辆空调公交车，争创国家公交示范城。加快智慧城市、信息惠民城市和海绵城市建设。按照中等城市标准，加强县城市政公共服务设施建设，积极引进建设一批城市综合体、商贸市场和医院学校。三是提升城市形象。加强阜城水系治理，重点整治中清河、西清河等18条城市内河水系。持续开展园林绿化提升行动，有效实施一批重点园林绿化工程，新增绿地面积410万平方米。按照“拆破旧、增绿量、强功能、惠民生”的要求，大力推进全面排查、综合整治、立体提升、精细管理，不断把“三项整治”向纵深推进。加大城市管理改革创新力度，提高环卫保洁覆盖面和标准，加快园林绿化、市政设施管理市场化改革进程。加强城市交通秩序整治，优化功能布局，分流纾解主城区交通压力。推动美丽乡村建设“以点为主”向“由点到面”的战略转换，全面推进美丽乡镇、中心村建设和自然村环境整治，重点抓好乡镇驻地中心村建设，扎实开展界首市整市推进美丽乡村建设省级试点工作，进一步改善人居环境。四是强化要素保障。充分发挥规划引领约束作用，抓紧完善相关专项规划，合理安排建设次序，严格规划管控，促进城市建设科学有序推进。继续把征迁安置工作作为城市建设的重中之重，重点抓好86个安置房和棚户区改造项目。用好城乡建设用地增减挂钩等政策，积极争取省预留计划指标，保障城市建设用地需求。加大土地收储出让力度，运用股权融资、项目融资等模式，积极发行城市建设债券，多渠道筹集建设资金，确保城市建设需要。

（四）突出改革攻坚，切实破除体制机制障碍。坚持把改革作为破解发展难题的关键举措，深化重点领域和关键环节改革，全面激发发展活力。一是积极推进供给侧结构性改革。认真贯彻宏观调控政策，开展“三去一降一补”行动，重点抓好产业结构调整、产品质量提升、降低企业成本、增加企业效益和加强金融监管、防范金融风险等工作。注重长远谋划，分析市场预期，科学有效调控，促进房地产市场平稳健康发展。二是深入推进行政体制改革。加快行政体制改革步伐，协同推进“放管服”，建设标准统一、资源共享的清单运行平台。推广“互联网+政务服务”，实现“数据多跑路、群众少跑腿”。加快公共资源交易监督管理体制改革，切实提升招投标工作水准。巩固扩大商事制度改革成果，开展注册登记电子化试点。三是扎实推进农村综合改革。深化农村集体土地制度改革，基本完成农村土地承包经营权确权登记颁证工作，加快集体土地所有权、农村集体建设用地、宅基地使用权确权登记颁证全覆盖。稳步推进农村宅基地管理制度改革，探索宅基地应保尽保制度和有偿退出机制。加快建立统一规范的农村产权交易平台，把沉睡的资产变成流动的资本。推动政策性农业保险提标扩面，探索建立农业风险分担机制。加强农田水利改革工作，创新小型水利工程建管体制。四是加快推进财税金融改革。建立全面规范、公开透明的预算制度，完善地方政府举债融资机制，加强财政资金绩效管理，提高资金使用效益。深化税收征管体制改革，落实税制改革任务。推动农商行建立健全现代商业银行制度，积极引进一批股份制商业银行。加快发展消费金融、商业保险、农民专业合作社资金互助社等非银行金融机构，着力提升金融服务中小微企业和支持农村发展的能力。大力推动企业上市挂牌。同时，按照一企一策、分类推进的原则，加快国有企业改革，确保国有资产效益最大化和效率最优化。

（五）突出创业创新，加快推进产业转型升级。坚持把调结构转方式促升级作为经济发展的永恒主题，深入实施创新驱动战略，有效激发大众创业、万众创新活力。一是切实加快“调转促”步伐。坚持把调转促“576”行动计划作为加快发展的重要抓手，健全完善调转促项目库，明确年度工作重点、具体工程项目和实施责任主体，做到有专人抓、有施工图、有时间表，全面推动各项任务落实。认真抓好太和现代医药产业集聚发展基地建设，积极争取全省第二批产业集聚基地。二是切实提升科技创新能力。强化企业创新主体作用，加强引导扶持，鼓励企业创新管理方式、加大研发投入，不断提升自主创新能力和产品开发水平，力争高新技术企业、省级创新型试点企业分别突破100家。完善产学研合作机制，推动企业与高等院校、科研院所开展联合攻关，加快技术成果转化。三是切实推进“双创”工作。大力实施“3611”创业行动计划和“大学生创业引领”计划，认真落实创业就业扶持政策，支持个体工商户转型升级创业，催生一批创业创新市场主体。支持专业技术人才创业，努力引进一批创业创新团队，发展壮大一批“创客新星”和“创业之星”。大力实施人才高地建设工程，积极引进各类优秀人才，注重培养技能型、实用型专业人才，加快“人口红利”向“人才红利”转化。

（六）突出精准扶贫，有效加快脱贫攻坚步伐。进一步加大力度，综合施策，坚决打赢脱贫攻坚战。一是大力推进“三项工程”。抢抓省委、省政府支持贫困地区基础设施建设的难得机遇，切实把水、电、路“三项工程”作为脱贫攻坚的突破口，明确年度计划，分解细化任务，落实配套资金，强化推进措施，加强质量监管，着力做好补齐短板大文章，努力提高贫困地区基础设施水平。二是有效实施专项扶贫。建立完善贫困户信息动态管理系统，精准掌握扶贫对象及贫困情况，确保一户不漏、一户不错。坚持整村推进、分类指导、因户施策，逐村逐户制定脱贫计划，分别采取产业扶贫、易地搬迁扶贫、医疗救助扶贫和社保兜底扶贫等办法，力争年内贫困人口减少20万左右。三是层层落实脱贫责任。切实把脱贫攻坚工作摆上重要位置，强化责任落实，纳入领导班子和领导干部目标考核体系。深入开展单位包村、干部包户定点帮扶工作，推进驻村扶贫工作制度化、规范化。加大市、县财政扶贫投入，推进扶贫开发投资主体多元化，鼓励各类企业、社会组织和个人参与扶贫工作。

（七）突出环境治理，着力加强生态阜阳建设。坚持把生态文明建设贯穿于经济社会发展的全过程，推进绿色低碳循环发展，建设天更蓝、地更绿、水更清的生态阜阳。一是大力开展造林行动。围绕创建国家森林城市和园林城市，持续加大投入力度，层层落实任务，重点抓好城市、村庄、田间地头和国省干道、县乡公路沿线造林绿化工作，确保全年新增成片造林6.8万亩、农田林网50万亩。认真筹办好省第七届花博会。二是大力开展治水行动。科学编制水资源利用规划，按照多用地表水、涵养地下水、治理污染水、节约使用水、保障三生水“五水同治”的要求，着力解决水多、水少、水脏的问题。加强水污染防治，加快城镇污水处理设施建设，推进淮河、济河、黑茨河等河流污染综合治理，促进工业污染源全面达标排放。大力推进治淮工程建设，加快南照淮河取水工程建设进度，实施行蓄洪区调整、洼地治理等项目，改造提升一批小型水利工程。三是大力开展治污行动。深入实施“颍淮蓝天工程”，继续抓好秸秆禁烧和综合利用，减少城市扬尘污染，基本淘汰黄标车，持续改善空气质量。启动土壤污染防治工作，实施化肥、农药零增长行动，着力解决土壤污染威胁农产品安全和人居环境健康问题。四是大力发展循环经济。以“减量化、再利用、资源化”为原则，扎实推进种植养殖循环发展、有色金属综合利用、煤化工节能技术应用等“十大重点工程”，推动循环经济向更高层次、更广领域迈进。实施全民节能行动计划，强化重点领域和重点用能单位管理。突出抓好泉河生态经济示范带建设，高起点、高标准编制总体规划，谋划实施一批农业综合开发、生态环境保护、高效产业发展等项目，努力建设成为全市生态人居示范区、绿色发展先行区。

（八）突出民生优先，努力维护社会和谐稳定。坚持以人为本，大力推进基本公共服务均等化，让发展成果更多更公平地惠及全体人民。一是切实保障和改善民生。按照省政府统一部署，明确33项民生工程内容，落实配套资金，加快实施进度，切实提升工程质量和效益。坚持把稳定就业作为经济发展的优先目标，重点做好农村劳动力、高校毕业生、城镇就业困难人员的就业工作。进一步深化“接您回家”活动，制定落实扶持政策，鼓励农民工返乡创业，引导农民工就地就业。进一步完善社会保障体系，全面实施机关事业单位养老保险制度，规范基本医疗、失业和生育保险市级统筹。新增各类保障性安居工程44626套，基本建成21033套。积极推行“房票”安置模式，提高货币化补偿安置比例。二是统筹发展各项社会事业。坚持教育优先发展战略，改善义务教育办学条件，开工建设阜阳一中、七中等新校区，大力发展职业教育，加快阜阳幼儿师范高等专科学校建设。完善医疗卫生服务体系，加强社区医疗服务中心建设。发展中医药事业。加快推进“医养结合”，着力提升养老服务水平。实施好全面两孩政策，提升计划生育服务管理水平，促进人口长期均衡发展。推进公共文化服务体系建设，办好省第四届全民健身运动会。加快祥源文化旅游区和八里河、颍州西湖等景区建设，强化市场开拓和产品开发，着力把阜阳建设成为区域旅游目的地。三是全面提高社会治理水平。坚持重大决策社会稳定风险评估制度，完善社会矛盾排查预警和多元化解机制。强化依法治访，切实解决群众合理诉求。深入开展“铸安”行动，有效防范和坚决遏制重特大事故的发生。落实农产品市场准入制度，强化食品、药品质量全过程监管，积极创建食品药品安全示范城市。加强应急平台和专业队伍建设，提高应急保障能力。进一步完善社会治安防控体系，严厉打击各类违法犯罪活动，持续加大毒品整治力度，提升人民群众安全感和满意度。强化统计工作基层基础。加强国防动员、后备力量建设和人民防空工作，落实优抚安置政策，促进军民融合深度发展，积极争创全国双拥模范城。高度重视国家安全、保密工作。支持工会、共青团、妇联和关工委、老龄委工作。做好档案、地震、气象、地名、地方志和民族、宗教、外事、侨务、港澳台等工作。发展红十字、残疾人和福利慈善事业。

各位代表！推动新常态下新发展、创造“十三五”新辉煌，对政府自身建设提出了更新更高的要求。我们要勇担历史使命，顺应群众期待，以忠诚、干净、担当为座右铭，以“三严三实”为行为准则，全面提升政府工作水平。一要保持坚强政治定力。严守政治纪律、政治规矩，坚定不移向中央看齐，始终与以习近平同志为核心的党中央保持高度一致，切实增强政治定力，做到政治上坚定自信、思想上同心同向、行动上高度自觉。各级政府要切实增强党性观念，自觉在党委领导下开展工作，认真落实党委决策部署，更好地推动政府工作。二要提升科学发展能力。牢固树立创新、协调、绿色、开放、共享的发展理念，主动学习政策、研究政策、用好政策。积极探索新常态下的新思路、新措施、新方法，善于把上级精神、外地经验同本地实际结合起来，善于抓大事、出实招、解难题，不断提高引领发展的能力，努力开创各项工作新局面。三要全面推进依法行政。坚持“法定职责必须为、法无授权不可为”，加强法治政府建设，严格按照法定权力和程序履行职能。着力规范政府系统决策行为，推进决策科学化、民主化、规范化。各级政府要自觉接受人大法律监督、政协民主监督，认真做好人大代表建议和政协委员提案办理工作，广泛听取民主党派、工商联、无党派人士和人民团体的意见，主动接受社会监督、舆论监督。深入推进政务公开，确保权力在阳光下运行。四要全心全意做好服务。从严从实抓好“三严三实”专题教育整改问题落实，确保善始善终、善作善成。健全政府领导干部联系基层工作机制，努力接足地气，尽心竭力为群众办实事。大力发展电子政务，推进网络问政和网上办事，进一步畅通联系服务群众的“最后一公里”。大力整治“庸、懒、散、拖”，坚决纠正不作为、慢作为、乱作为，不断提高行政效能。五要着力打造廉洁政府。全体政府工作人员尤其是领导干部要模范遵守廉洁自律准则，自觉廉洁从政、廉洁用权、廉洁修身、廉洁齐家，恪守道德高线和纪律底线。严格履行“一岗双责”，加强行政监察和审计监督，把权力关进制度的笼子。严肃查处各类违法违纪案件，加大反腐倡廉力度，不断压缩腐败现象生存空间。驰而不息改进作风，严防“四风”反弹，厉行勤俭节约，着力打造为民务实清廉政府。

各位代表！辉煌成就鼓舞人心，宏伟蓝图催人奋进。让我们紧密团结在以习近平同志为核心的党中央周围，在中共阜阳市委的正确领导下，锐意进取，开拓创新，扎实工作，努力开创全市经济社会发展新局面，为全面建成小康社会而努力奋斗!
